# Left atrial wall shear stress correlates with fibrosis in patients with atrial fibrillation

**DOI:** 10.1038/s44161-025-00651-z

**Published:** 2025-05-13

**Authors:** Dionysios Adamopoulos, Georgios Rovas, Nicolas Johner, Hajo Müller, Jean-François Deux, Lindsey A. Crowe, Jean-Paul Vallée, François Mach, Nikolaos Stergiopulos, Dipen Shah

**Affiliations:** 1 Division of Cardiology, Department of Internal Medicine, Hôpitaux Universitaires de Genève, Geneva, Switzerland; 2 Department of Medicine, Faculty of Medicine, Geneva University, Geneva, Switzerland; 3 Division of Nuclear Medicine, Department of Diagnostics, Hôpitaux Universitaires de Genève, Geneva, Switzerland; 4 Laboratory of Hemodynamics and Cardiovascular Technology, École Polytechnique Fédérale de Lausanne, Lausanne, Switzerland; 5 Division of Radiology, Department of Diagnostics, Hôpitaux Universitaires de Genève, Geneva, Switzerland

**Keywords:** Atrial fibrillation, Blood flow, Biomedical engineering

## Abstract

Left atrial wall fibrosis has an important role in atrial fibrillation (AF) because of the abnormal electrophysiological properties of the fibrotic areas. However, the mechanisms behind the development of left atrial fibrosis are not well understood. Here, we examine the association between regional wall shear stress and areas with fibrosis in the left atrium of patients with AF. We recruited 15 patients with AF for an observational prospective study involving baseline three-dimensional (3D) electroanatomical mapping of the left atrium and preinterventional cardiovascular magnetic resonance imaging to detect left atrial fibrosis. We extracted a 3D anatomical model of the left atrium from the electroanatomical maps. Then, we calculated regional time-averaged wall shear stress (TAWSS) and blood stagnation by performing patient-specific computational fluid dynamic simulations. We found that fibrosis and electrical scarring were more prevalent in areas exposed to high TAWSS without blood stagnation, whereas areas with low TAWSS were associated with blood stagnation.

## Abstract (web summary)

Adamopoulos, Rovas et al. show that high regional wall shear stress correlates with fibrosis and electrical scarring in the left atrium of patients with atrial fibrillation, providing insight into the development of fibrotic tissue.

## Main

Atrial fibrillation (AF) is the most frequent sustained arrhythmia worldwide, with notable morbidity and mortality rates, especially in older adults. In the Global Burden of Disease 2010 study, AF was estimated to affect approximately 33 million individuals globally and was found to be strongly associated with cerebrovascular events, heart failure, hospitalizations and death^1^.

The presence of fibrotic areas in the left atrial wall has a pivotal role in the perpetuation of AF^2^. It is now widely accepted that the pathophysiology of AF comprises two distinct (but equally important) mechanisms: (1) the presence of multiple rapidly firing ectopic foci found principally around the pulmonary veins, which act as triggers initiating the arrhythmia^3^, and (2) the presence of atrial myocardial cells with impaired electrical conductivity (electrical scars) and/or abnormal repolarization characteristics, which are capable of sustaining the arrhythmia through the formation of pathological electrical wavelets, reentry circuits and abnormal electrical impulses^4^. Although the trigger component of AF has been extensively studied, little is known about the mechanisms by which electrical scars are created, leading to the perpetuation of the arrhythmia.

Left atrial myocardial cells are constantly exposed to the hydrodynamic forces of the blood flow arriving from the pulmonary veins. In particular, wall shear stress (WSS; defined as the frictional force per unit area exerted by the blood flow tangentially on the wall) has been recognized as a major hemodynamic factor affecting both the function and geometry of different parts of the cardiovascular system^5^. More precisely, the exposure of the arterial wall to pathological (both high and low) shear stress has been consistently associated with progressive vascular damage, including atherosclerosis and aneurysm growth and rupture, through both inflammatory cell- and mural cell-mediated pathways^6^. Indeed, the exposure of the arterial wall to high WSS has been shown to initiate biochemical cascades (through endothelial cell mechanotransduction) that lead to local production of proteases and, finally, apoptosis of the wall’s smooth muscle cells^7^.

Based on the above-mentioned observations, we hypothesized that exposure to high shear stress may have a role in the development of fibrotic areas in the left atrial wall. While prior studies have suggested apoptosis as a potential mechanistic link between shear stress and fibrosis, our study focuses on examining the association between regional WSS and fibrotic areas exhibiting impaired endocardial voltages in the left atrial wall of patients with AF.

## Results

### Patient population and clinical presentation

**Fig. 1:**
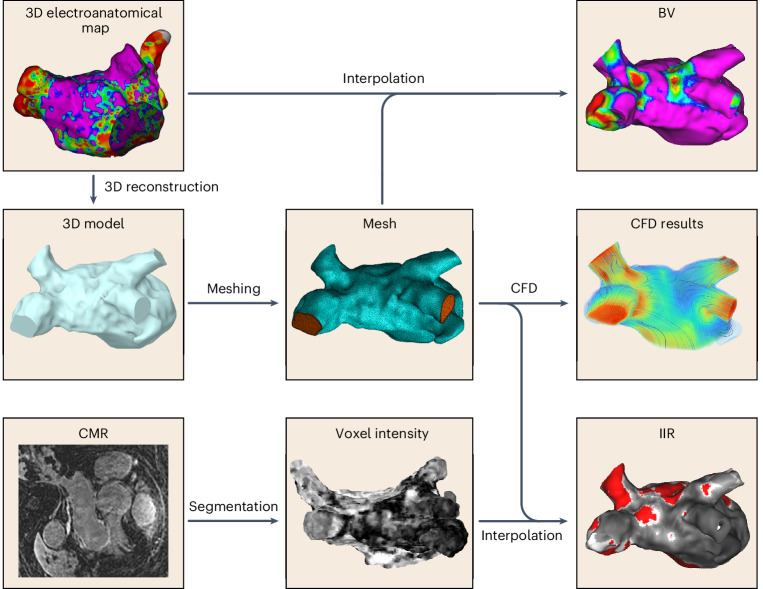
Graphical overview of the methodology. The diagram outlines the methodological steps, integrating data from the electroanatomical maps (left atrial geometry and BV), CFD analysis and CMR imaging with gadolinium contrast.

**Fig. 2:**
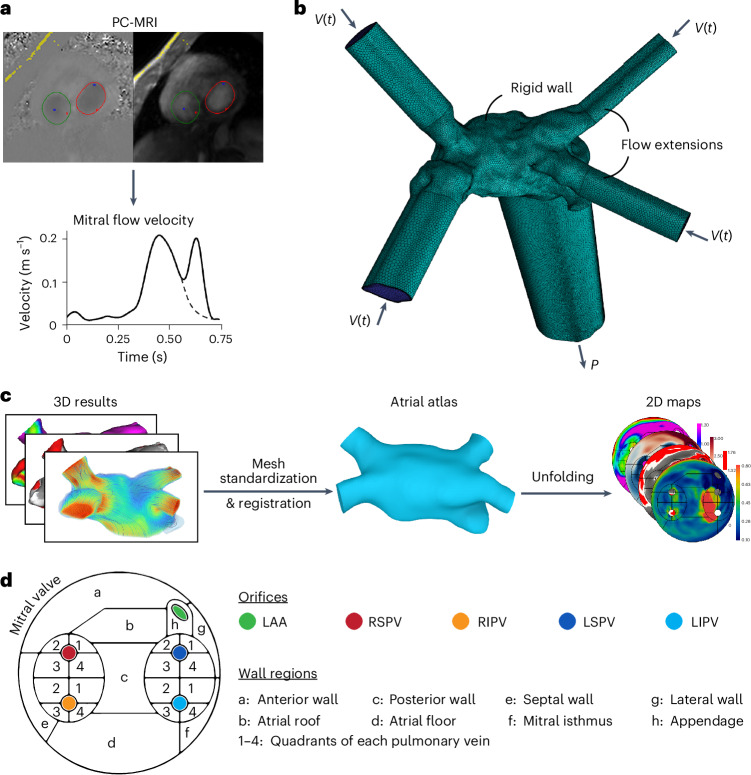
CFD and postprocessing methodological overview. **a**, Derivation of patient-specific mitral flow velocity from phase-contrast CMR imaging with adaptation to an AF mitral velocity waveform (dashed line) by removing the A-wave, if required. **b**, Example of the boundary conditions and flow extensions on a 3D mesh. **c**, Methodological steps of the unfolding procedure to generate the 2D maps through a standardized atrial atlas. **d**, Regions of the 2D atrial maps. PC-MRI, phase-contrast MRI; *V*(*t*), time-dependent flow velocity; *P*, pressure; LAA, left atrial appendage; RSPV, right superior pulmonary vein; RIPV, right inferior pulmonary vein; LSPV, left superior pulmonary vein; LIPV, left inferior pulmonary vein.

We prospectively recruited patients who had symptomatic episodes of AF with a clinical indication for catheter ablation (*n* = 15, 13 men, mean age 61 ± 11 years). The study population included individuals who presented with paroxysmal AF (*n* = 10), persistent AF (*n* = 5) and atrial flutter (*n* = 3). All patients underwent cardiovascular magnetic resonance (CMR) imaging to derive the image intensity ratio (IIR), an index of fibrosis, and three-dimensional (3D) electroanatomical mapping during the catheter ablation procedure to derive the bipolar voltage (BV), an index of electrical scarring. The 3D atrial geometry was extracted from the 3D electroanatomical maps to generate a 3D mesh, which was then used for patient-specific computational fluid dynamic (CFD) simulations of the left atrium (Figs. 1 and 2a,b). From the simulations, we computed the distribution of the time-averaged WSS (TAWSS), the blood age (BA) and other hemodynamic indices on the atrial wall. We projected all variables onto the CFD mesh of each patient (Fig. 1) and then unfolded the atrial wall into a 2D map through a standardization and registration procedure (Fig. 2c) to allow for statistical comparisons and to divide the atrial wall into standardized regions (Fig. 2d).

### Regional distribution of TAWSS

**Fig. 3:**
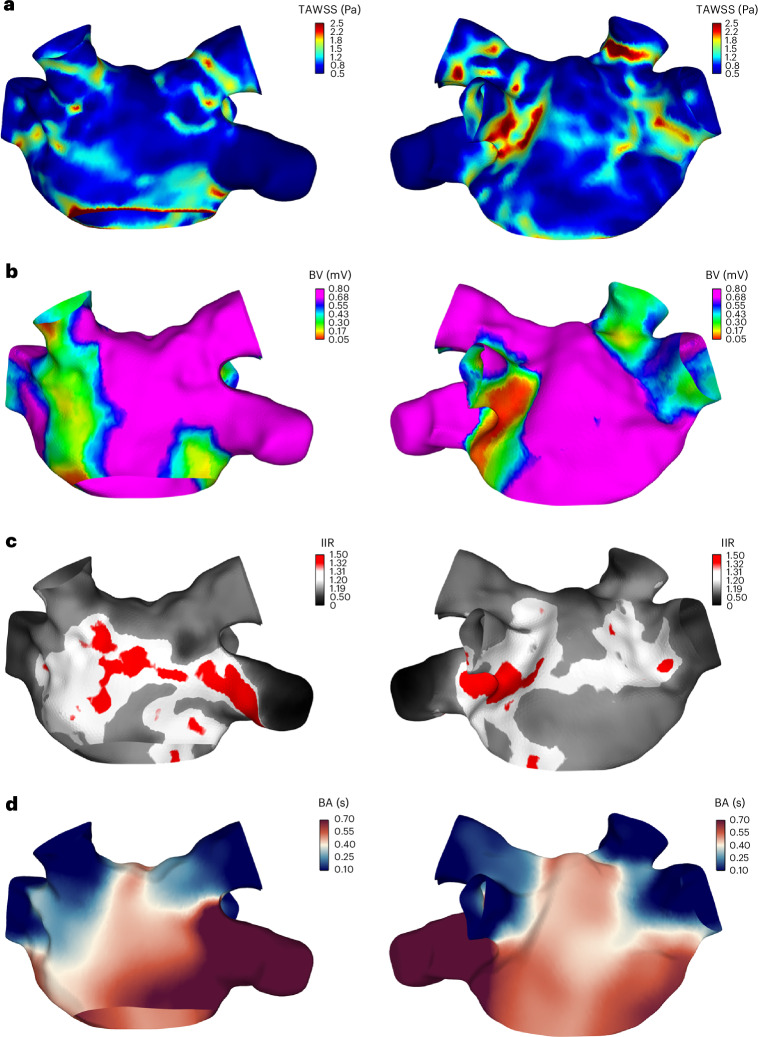
Three-dimensional results in an exemplary case. **a**–**d**, Distribution of wall shear and fibrosis on the 3D atrial model of participant 10 in anteroposterior (left) and posteroanterior (right) views. The colors represent the TAWSS (**a**); the BV (**b**); the IIR (**c**) in regions with fibrosis (red), interstitial fibrosis (white) and no fibrosis (gray); and the BA (**d**). Source data

The absolute values of TAWSS varied among participants depending on the size and anatomy of each atrium and the cardiac output. The distribution of TAWSS is illustrated on a 3D model for an exemplary case in Fig. 3 and for all cases in the 2D unfolded maps in Extended Data Fig. 3. The pulmonary veins had significantly higher TAWSS than the rest of the atrium (*P* = 0.003). In all cases, at least one high-TAWSS region was observed near the pulmonary vein ostia. Interestingly, the left pulmonary veins had 22% higher TAWSS compared to the right ones (*P* < 0.001).

### Regional distribution of fibrosis markers

**Extended Data Fig. 1:**
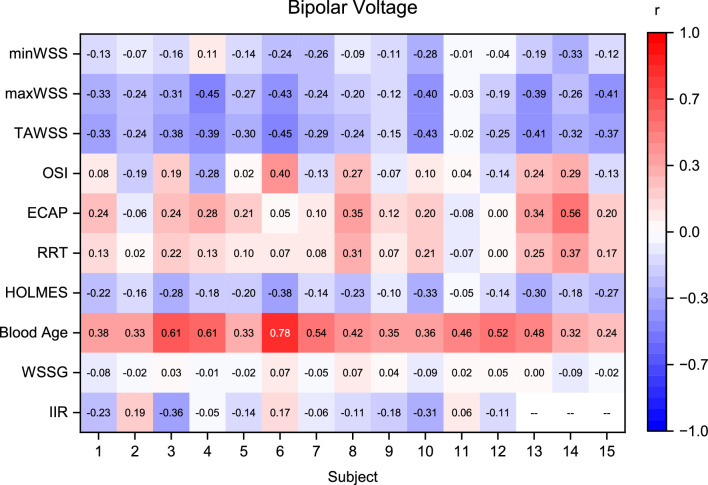
The connection between electrical scar and hemodynamic indices. Pearson’s correlation coefficient heatmap between the bipolar voltage and the calculated hemodynamic indices of each subject. The 3D electro-anatomical maps of subjects 2, 3, 5, 9, 10, 11 and 14 were obtained in atrial fibrillation. Source data

**Extended Data Fig. 2:**
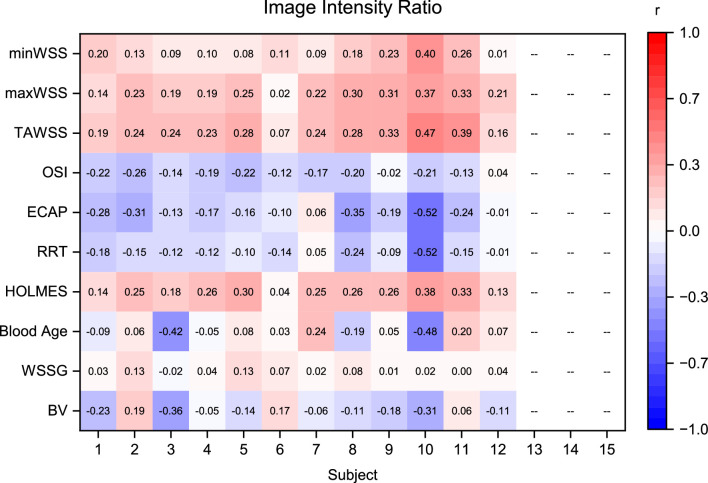
The connection between fibrosis and hemodynamic indices. Pearson’s correlation coefficient heatmap between the image intensity ratio and the calculated hemodynamic indices of each subject. Source data

**Extended Data Fig. 3:**
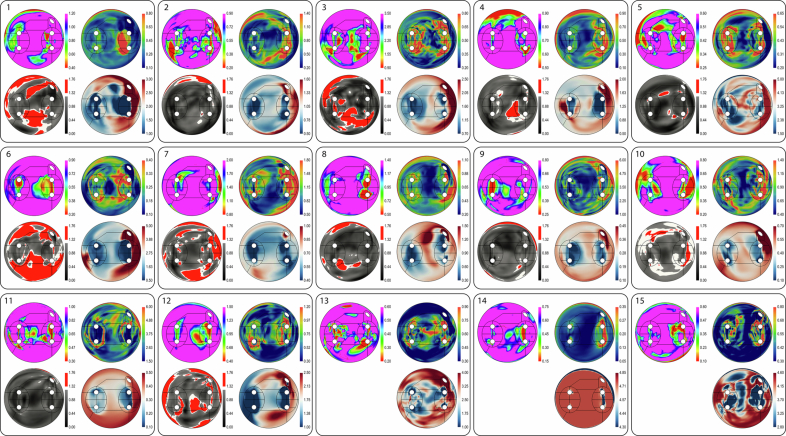
The 2D maps with the results of all subjects. The 2D maps of the distribution of the time-averaged wall shear stress (TAWSS), bipolar voltage (BV), blood age (BA) and Image Intensity Ratio (IIR) for each subject. The colormap of IIR corresponds to healthy regions (black-gray), regions with interstitial fibrosis (white) and fibrotic regions (red). LGE-CMR acquisitions were not available for subjects 13–15.

A representative case of the 3D distribution of the electrical scar and CMR fibrosis on the atrial wall is presented in Fig. 3. The 2D atrial maps of the BV and IIR for all participants are demonstrated in Extended Data Fig. 3. In most of the cases, areas with lower BV were more frequently observed at the roof of the atrium and around the pulmonary veins (Extended Data Fig. 3). Moreover, in most of the cases (*n* = 9), a negative association was observed between BV and IIR (Extended Data Figs. 1 and 2). The inverse relationship was noticed in three cases with very low (participants 2 and 11) and very high (participant 6) levels of CMR fibrosis (Extended Data Figs. 1 and 2).

### The interplay between hemodynamics and fibrosis

**Fig. 4:**
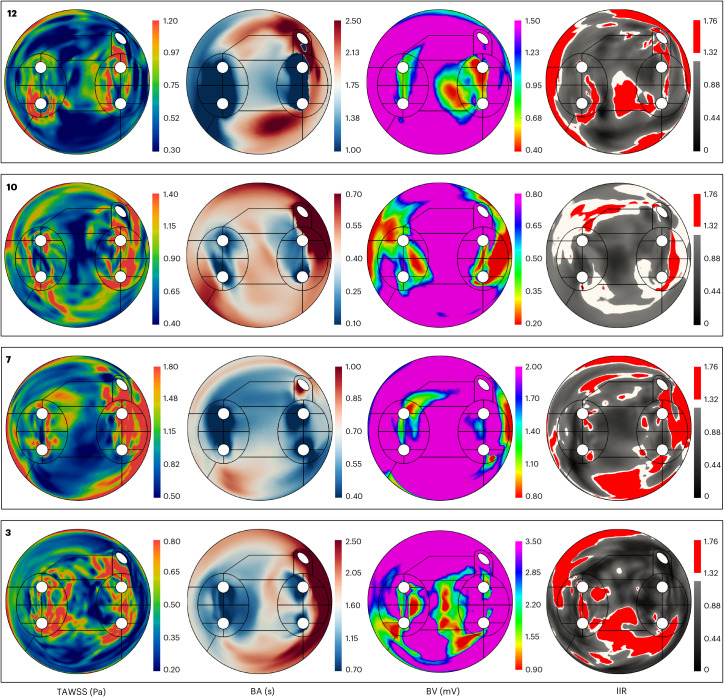
Combined 2D maps of four representative cases of the primary variables: TAWSS, BA, BV and IIR. Each panel corresponds to one participant denoted by the corresponding participant number.

There was a notable overlap in regions with low BV, elevated TAWSS and high IIR, as can be seen for four representative cases (Fig. 4). TAWSS was found to have a significant negative correlation with BV in all cases (*n* = 15, Pearson *r* ranging from −0.021 to −0.449, case 11: *P* = 0.003, all other cases: *P* < 0.001) and a significant positive correlation with IIR (*n* = 12, Pearson *r* ranging from 0.071 to 0.475, all cases: *P* < 0.001). The AF type (paroxysmal or persistent) did not significantly alter the correlation of TAWSS with BV (*P* = 0.5) and IIR (*P* = 0.49) or the correlation of BV with IIR (*P* = 0.53). Regarding their correlation with fibrosis and electrical scarring, the other shear stress indices that we evaluated had (1) nonsignificant correlations, (2) a good correlation with only one of the two measures of fibrosis, (3) lower correlations compared to TAWSS or (4) inconsistent (both positive and negative) correlations, depending on the participant (Extended Data Figs. 1 and 2).

**Fig. 5:**
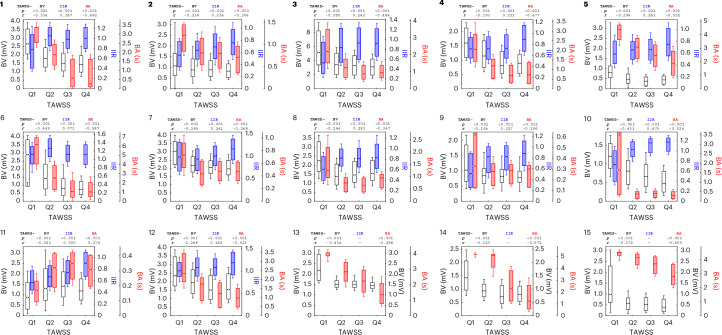
Participant-specific interplay between TAWSS, BA and fibrosis as measured using BV and IIR. The TAWSS is divided into quartiles (Q1–Q4). Above each chart is the corresponding two-sided *P* value from the Kruskal–Wallis test between the quartiles of TAWSS and BV, IIR or BA and the Pearson correlation coefficient (*r*) between all data points of the same variable pairs. The boxes correspond to the interquartile range, the notches to the 95% confidence interval of the median and the whiskers to the s.d. LGE-CMR acquisitions were not available for participants 13–15. Sample sizes: 1: 29,492, 2: 27,780, 3: 37,670, 4: 23,899, 5: 28,750, 6: 28,371, 7: 18,797, 8: 19,376, 9: 23,103, 10: 20,840, 11: 20,444, 12: 22,615, 13: 30,219, 14: 21,990 and 15: 35,771.

**Fig. 6:**
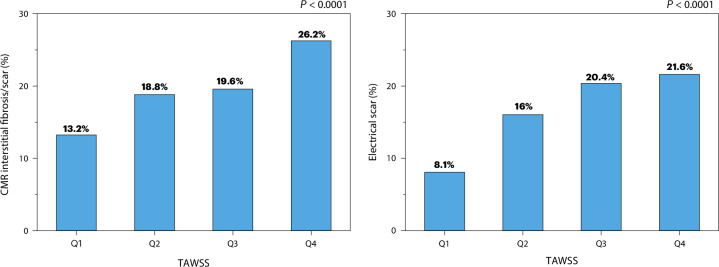
Relationship between fibrosis distribution and TAWSS. CMR-derived fibrosis (left; IIR ≥ 1.2, *n* = 301,137) and electrical scar (right; BV < 0.5 mV, *n* = 389,117) data are shown according to the TAWSS quartiles for the total study population, with the corresponding two-sided *P* value from the chi-square test.

To quantify the relationship between WSS and fibrosis further, we divided the TAWSS values into four quartiles and calculated the BV and IIR of each quartile for each participant (Fig. 5). We observed a consistent trend for both BV and IIR with increasing TAWSS in all cases, although to different extents. The means of BV and IIR of each quartile were found to be significantly different in all cases (*P* < 0.001). Finally, regions with higher TAWSS exhibited higher degrees of fibrosis and electrical scarring as detected by both low BV and high IIR (*P* < 0.001), according to the standard diagnostic criteria^8,9^ (Fig. 6).

### Regional distribution of BA

**Fig. 7:**
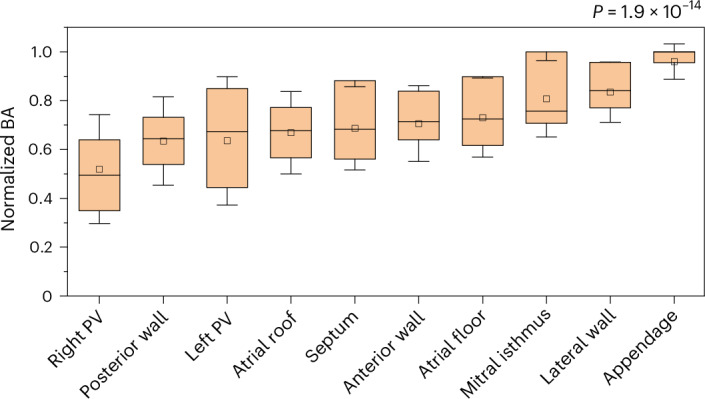
Distribution of BA in the left atrial regions. Data show the normalized BA according to the left atrial regions of the total study population. The *P* value corresponds to the result of the two-sided Kruskal–Wallis test (*n* = 60, for the pulmonary veins, *n* = 15 for all other regions). The boxes correspond to the median and the interquartile range, the whiskers to the s.d. and the square to the mean value. PV, pulmonary vein. Source data

Differences in the regional distribution of BA were noted in all study participants (Fig. 7; *P* < 0.001). The longest BA duration was observed consistently in the left atrial appendage, with the shortest values in the pulmonary veins and at their ostial junction with the left atrium. A point-to-point comparison between TAWSS and BA showed strong, inverse correlations in all but one case (Pearson *r* ranging from −0.268 to −0.688, *P* < 0.001 for all, except case 11; Fig. 5 and Extended Data Fig. 3). BA also showed a strong positive correlation with BV in all cases (Pearson *r* ranging from 0.07 to 0.78, *P* < 0.001 for all; Extended Data Fig. 1).

## Discussion

This study attempted to associate hemodynamic indices, specifically WSS, with atrial wall fibrosis as assessed by both electrical mapping and CMR imaging^10^. The main findings of the present study can be summarized as follows: (1) in patients with AF, the regional distribution of fibrosis and electrical scarring as assessed by both BV and IIR followed specific patterns, with an important presence in the areas around the pulmonary veins and at the roof of the left atrium. (2) The same regional distribution was also observed for WSS, which showed significant correlations with the fibrosis and electrical scar indices. Consequently, in patients with AF, areas with high WSS exhibited more pronounced fibrosis and electrical scarring, pointing to a new pathophysiological link explaining the well-established association between AF and pathologies with an impaired left atrial hemodynamic environment (for example, systolic or diastolic left ventricular dysfunction, mitral regurgitation)^11,12^. (3) Finally, areas with high BA were not associated with areas with low BV or high IIR, suggesting that blood stasis and its biochemical by-products are not responsible for the development of fibrosis and electrical scarring. Many in vitro studies have focused on the cellular response of atrial myocytes to WSS. Boycott et al.^13^ reported that the application of shear stress on atrial myocytes generated an outward current mediated by K^+^ channels and resulted in a shortening of the action potential duration. The shear stress threshold for this shear-induced activation was reported to be 0.28 Pa, whereas dilated and fibrotic atria were shown to be less responsive to the shear-induced current increase, indicating that shear stress could primarily affect the early stages of AF. Son et al.^14^ showed that shear stress >0.2 Pa can induce a shear stress-sensitive current on the membrane of atrial myocytes and trigger the release of Ca^2+^ in the subsarcolemmal domains. In addition, Yamamoto et al.^15^ demonstrated that endothelial cells react to WSS through a mechanotransduction pathway. Specifically, it was shown that WSS as low as 0.1 Pa can increase ATP production in the mitochondria, followed by increased extracellular ATP release that increases the influx of extracellular Ca^2+^. Ca^2+^ release or influx favors abnormal impulse generation, likely as a triggered activity. Thus, these findings could well account for electrical remodeling, including shortened action potential durations as well as AF-initiating triggers. In other observations, Morel et al.^16^ showed that high WSS (>8 Pa) affected the shape and architecture of endothelial cells, upregulated the expression of proteins related to the cytoskeleton and downregulated extracellular matrix proteins. In our CFD simulations, the maximum WSS reached this threshold in all cases, whereas it ranged up to one order of magnitude higher in certain participants. Furthermore, Meng et al.^6^ showed that high WSS in combination with high WSS gradient (WSSG) can lead to endothelial cell damage and turnover, extracellular matrix degradation and mural cell apoptosis through cell-mediated mechanisms^6^. Finally, in a recent study by Oh et al.^17^, abnormal flow conditions characterized by high WSS (regurgitant flow) were associated with substantial endocardial fibrosis through endothelial-to-mesenchymal transition in a rodent model. These pathways provide a molecular background explaining the association between fibrotic remodeling and high WSS observed in our study.

The dependence of the degree of electrical scarring and fibrosis on TAWSS appeared to be nonlinear, rapidly increasing with increasing TAWSS and then plateauing (Fig. 5). Therefore, the Pearson linear correlation coefficient could not accurately summarize this behavior but served more as an indication of this relationship, prompting us to perform subsequent analyses in TAWSS quartiles. The presence of persistent AF did not alter the correlation of TAWSS with BV and IIR, indicating that a long history of AF and the consequent atrial wall remodeling might not be responsible for the elevated wall shear in those regions, although further longitudinal studies would be required to clarify the causality among wall shear, electrical scarring and fibrosis. TAWSS achieved good localization of affected regions, as can be confirmed by the visual comparison of the 2D maps especially for BV (Fig. 4 and Extended Data Fig. 3). The same is true for BA and BV, as expected given the direct connection of both TAWSS and BA with the velocity field, which is derived through the fluid dynamic equations. The fibrotic patterns, as evidenced by the BV maps, are replicated with good accuracy in the patterns of TAWSS, even in cases in which the statistical analysis does not clearly show this relationship (for example, case 11). In cases with high asymmetry of fibrosis between the left and right sides of the left atrium (for example, cases 8 and 14, which both showed increased fibrosis between the left superior and inferior pulmonary veins), the TAWSS results confirmed the observed asymmetry.

Several studies have examined the distribution of regional left atrial fibrosis in patients with AF. Caixal et al.^18^ recently reported that left atrial fibrosis was predominantly located in the area adjacent to the descending aorta. In the same direction, Khosknab et al.^19^ observed higher levels of left atrial wall fibrosis in areas near the aorta among ablation-naive patients. Finally, Zghaib et al.^20^ found a strong association between left atrial pericardial adipose tissue and electrical scarring of the wall. The authors attributed these findings to the repetitive impact of the distention–compression cycles of the aorta on the wall of the left atrium, provoking chronic wall stretch and/or the release of inflammatory cytokines locally. Our study provides another potential mechanism explaining these observations, as local left atrial wall deformation by extrinsic compression could increase WSS due to alterations in left atrial geometry.

This finding is of major practical importance as CMR-based estimations of TAWSS could become readily available in clinical routine, providing noninvasive information about potential areas and targets for ablation. This technique has been primarily used and validated against CFD or in vitro results in the aorta, arterial aneurysms and bifurcations, whereas CMR-derived wall shear indices have been shown to correlate with clinical biomarkers and adverse outcomes^21^. However, the derivation of wall shear from a velocity field is still the subject of ongoing research, and the existing methods still produce inaccurate results even with synthetic data on relatively simple geometries^22^. 4D flow CMR imaging has been used for the investigation and quantification of hemodynamic metrics in AF^23^, but the CMR-derived wall shear of the left atrium has not yet been validated and is subject to many limitations, including the insufficient resolution of CMR imaging and the complex geometry and flow patterns of the left atrium^24^. Because of these limitations, we decided to estimate wall shear indices following the standard approach, through CFD, despite the complexity and necessary assumptions. When CMR-based shear indices are validated and provide sufficient accuracy in the left atrium, the need for CFD analysis will become obsolete, and wall shear indices will be computed directly.

In the present study, CMR-derived fibrosis and areas with low endocardial voltage showed different levels of correlation, with the vast majority being negative (*n* = 9 (75%); Extended Data Figs. 1 and 2). Atrial fibrosis has been proposed as the main mechanism underlying low BV as it interrupts fiber bundle continuity, which leads to local conduction abnormalities^25^. However, point-by-point data assessing the agreement between late gadolinium enhancement (LGE)-CMR atrial wall intensity and BV are scarce. Harisson et al.^26^ found only weak associations between atrial wall signal intensity and endocardial voltage in patients undergoing redo procedures. In a recent study, LGE-CMR-derived atrial fibrosis as assessed using the IIR correlated with endocardial BV, with the relationship becoming weaker with atrial dilation^27^. These discrepancies may be, at least partially, explained by technical artifacts and interindividual variability. Moreover, it has been reported that BV depends on the electrophysiological conditions, including a strong dependence on atrial frequency, rhythm and activation wave direction^28^. Another possible explanation for the weaker correlation between atrial wall signal intensity and endocardial voltage may be that early-stage interstitial fibrosis is not detected by the LGE-CMR sequences^29^.

Despite the good agreement between TAWSS and fibrotic patterns near the pulmonary veins and posterior wall, not all fibrotic patches conformed to regions with high TAWSS. A common exception was exemplified by large fibrotic patches on the anterior wall and the atrial floor near the mitral valve, which were not matched by equally large areas with high TAWSS. This could be attributed to the effect of outgoing flow through the mitral valve on the local hemodynamics, the absence of a valve leaflet motion model in our simulations or the atrial compression caused by the aorta, as explained previously. Similar exceptions have also been recorded in previous studies^10^.

Consistent patterns in the regional distribution of BA were also noted in all participants, with the appendage presenting the highest blood stagnation index, followed by the lateral wall, mitral isthmus and atrial floor. On the contrary, areas around the ostia of the pulmonary veins, the posterior wall and the atrial roof presented lower BA (Fig. 7). As expected, an inverse association with TAWSS was observed as high velocities contribute to the rapid ‘cleansing’ of the blood from the different areas of the left atrium, whereas low velocities result in blood stagnation. This observation further highlights the utility of evaluating the left atrial TAWSS as it predicts not only the location of the AF substrate (highest end values) but also areas with high thrombogenic potential (lowest end values). This may contribute to a patient-specific thrombogenic risk assessment that extends beyond classic risk scores in the era of personalized medicine.

Unlike in other cardiovascular conditions, the use of CFD, structural or combined fluid–structure interaction simulations has been limited in AF, in terms of both the overall number of studies and the number of patients included in each study^30^. While computational approaches are gaining momentum as an additional diagnostic tool, their results in the left atrium and ventricle have been validated against 4D flow CMR^31^, and certain cardiac CFD methods have even received clinical approval^30^. Hunter et al.^32^ showed that regions with low voltage and electrical scarring have higher wall stress, through structural left atrial models imposed solely on the transmural pressure without accounting for the hemodynamics. CFD simulations have been previously used to calculate indices of WSS on the left atrial wall in the presence or absence of AF^33^ and to assess catheter ablation outcomes and the risk of thrombosis^34,35^, but in none of those cases have the results been compared to measurements of markers of fibrosis. Our results agree well with the reported ranges of hemodynamic indices in the left atrium of patients with AF in those studies.

WSS indices have been compared in fibrotic and nonfibrotic left atrial regions of patients with AF^10^. Although the reported ranges of the shear indices are similar to those presented here, our findings disagree with the distribution of those indices in healthy and fibrotic regions. This discrepancy could be attributed to certain methodological differences as the authors of the previous study used (1) CMR imaging to derive the CFD model, which has lower spatial resolution and results in smoother model surfaces; (2) generic boundary conditions and generic wall motion; (3) only CMR to identify fibrotic areas, which has lower resolution and specificity compared to BV; and (4) a fewer number of patients. Furthermore, the previous study’s limitation of providing solely summarized data, without the regional distribution of shear indices on the left atrial walls, hindered the possibility of conducting more comprehensive comparisons aimed at identifying additional reasons for the observed differences.

### Limitations

In our study, WSS was estimated based on CFD methods and was not measured directly. The CFD methodology is based on assumptions and the application of boundary conditions that may markedly affect the results, although to a small extent. Specifically, the most important assumptions that have been made in this study are the absence of mitral valve leaflets, the stationary atrial wall, the absence of separate time-dependent flow inlets at each pulmonary vein and the laminar flow. All these assumptions have been compared against more complex models that circumvent them, and they were found to be reasonable with only minimal impact on the solution^36^. The only exception was blood residence-related indices of the left atrial appendage, where stationary-wall and moving-wall models produced different results, but this region is not included in the present results.

The interaction of the atrial wall and mitral valve leaflet motion with the blood has not been considered in our CFD simulations. The validity of the model could be improved if these assumptions are rendered obsolete, but that would require dynamic imaging and the use of a fluid–structure interaction methodology instead of CFD. Further research would also be necessary to investigate whether the added complexity of such methodology is justified by the gain in accuracy, taking into account the resolution limitations of dynamic imaging techniques. Due to the selected methodology, we decided to exclude patients with considerable mitral regurgitation, as the CFD model was unable to capture regurgitation jets accurately, which could alter the atrial hemodynamics. The effects of fixed or moving walls on the left atrial hemodynamics have been studied previously^37^. The authors compared the influence of fixed-wall and moving-wall models, albeit with slightly different inlet boundary conditions, on atrial flow patterns, blood residence time and kinetic energy but not on wall shear indices. They concluded that the incorporation of wall motion affected mostly the flow fields of atria with normal contraction and, to a lesser extent, fibrillating atria due to the limited and difficult-to-model wall motion in the latter case. Considering these findings and due to the absence of patient-specific time-dependent imaging data, we decided to model the wall as stationary in our simulations.

Further, instead of imposing the same velocity profile on the pulmonary veins, their flow could be measured and imposed independently while simultaneously imposing an outlet velocity profile at the mitral valve and a time-dependent outlet pressure. As in the moving-wall case, these boundary conditions will necessitate the use of fluid–structure interaction models and additional measurement by echocardiography or phase-contrast CMR imaging.

We assumed laminar flow, which was a reasonable assumption based on the Reynolds number, but turbulence could occur locally due to partial mitral valve opening, valve leaflet motion, wall motion and simultaneous inflow from multiple directions. This choice was guided by the computational complexity of turbulence models, the fact that the chosen blood model is incompatible with turbulence and the additional parameters required by turbulence models, which are difficult to estimate. Before choosing the flow model, we conducted a comparison of laminar and turbulent flow in a single case and, similar to Dueñas-Pamplona et al.^36^, we did not observe differences in the results that could justify the use of turbulence models, given their added complexity.

Although the results are statistically significant and the conclusions are clinically relevant, the small sample size should be acknowledged as a limiting factor and the results may not be generalizable to all patients with AF. Eight patients in our study underwent BV mapping in sinus rhythm and the remaining patients during AF; however, a good correlation has been reported between BV measured in sinus rhythm versus during AF^38^. Pertinently, a sensitivity analysis in our cohort showed that the correlation of BV with TAWSS, BA and IIR was maintained and similar whether the electroanatomical map was acquired during AF or sinus rhythm. In addition, the results may not be applicable to the general population as specific inclusion criteria and CMR demands were applied. Finally, although most of the processes were semiautomated and operator-independent, the segmentation was still performed manually.

## Conclusions

We conclude that left atrial wall regions with CMR markers of fibrosis and low endocardial voltage notably overlap with regions of increased WSS, and they develop preferably near the pulmonary veins and the atrial roof. Contrarily, areas prone to blood stagnation with high BA were not associated with fibrosis or impaired endocardial voltage. These results are supported by patient-specific simulations of the hemodynamic conditions in the left atrium, and they suggest a potential pathophysiological link between AF and a disturbed hemodynamic environment. Further developments in CMR-based wall shear measurement and automated CFD workflows of the left atrium could allow the noninvasive localization of regions prone to AF substrate development and regional stasis.

## Methods

### Study population

All patients underwent baseline echocardiography, and only those without hemodynamically considerable (more than moderate) mitral regurgitation were eligible for the study. In addition, only patients without contraindications to CMR imaging (for example, metallic implants) and with overall health that allowed them to undergo an extended CMR examination were eligible for the study. No participant presented with pronounced coronary artery disease at the time of study inclusion. Data were anonymized before analysis. Written informed consent was obtained from each participant for the use, analysis and publication of their data for research purposes. The study protocol was approved by the Commission Cantonale d’Éthique de la Recherche sur l’être humain (CCER) of the canton of Geneva, Switzerland (approval no. 2023-02314).

### CMR imaging

#### CMR acquisition protocol

All patients underwent CMR imaging at baseline before catheter ablation (5 ± 3 days before the ablation, ranging from the same day to 8 days before). All scans were performed using a 3-T clinical, ceiling-mounted, intraoperative magnetic resonance imaging (MRI) system (IMRIS, Deerfield Imaging) combined with the Artis electrophysiology suite (MAGNETOM Skyra/Artis, Siemens) using two 18-channel array coils (anterior and posterior). The CMR protocol included balanced steady-state free precession cine imaging in long-axis orientation (two-chamber, four-chamber and short-axis stack views). An electrocardiogram-gated free-breathing 3D contrast-enhanced magnetic resonance angiogram of the left atrium and the pulmonary veins was obtained immediately after contrast agent (Dotarem, Guerbet) injection. For left atrial fibrosis, a 3D LGE-CMR sequence was acquired 15 min later (Siemens prototype ‘whole heart’) with Dixon fat suppression and a 100% efficiency iNav respiratory motion navigator during a static period of the cardiac cycle in terms of atrial contraction. The typical acquisition parameters were as follows: repetition time, 10 ms; TE1 and TE2, 1.3 and 2.8 ms; flip angle, 25°; in-plane resolution, 1.3 × 1.3 mm with a slice thickness of 1.3 mm; acquisition time, 7 min 52 s ± 106 s. For the phase-contrast flow acquisition of the mitral valve, the parameters were as follows: TR/TE, 28/2.4 ms; in-plane resolution, 1.9 × 1.9 mm with a slice thickness of 6 mm; velocity-encoding value, 250 cm s^−1^; GRAPPA (generalized autocalibrating partially parallel acquisitions) acceleration factor, 2; acquisition time, 116 heartbeats.

#### CMR image analysis

The CMR images were obtained during ongoing AF in 2 participants and during sinus rhythm in 13 participants. Segmentation of the left atrium was performed in free and open-source software for image analysis (3D Slicer)^39^. Initially, the blood pool of the left atrium (including the pulmonary veins) was manually segmented on the CMR axial slices. A two-voxel surface dilation was used for delimiting the epicardial border, and a 3D model of the left atrial wall was created. The mitral annulus was used to separate the left atrium from the left ventricular cavity. On the 3D LGE-CMR images, the signal intensity was normalized to the mean blood pool intensity as described in the IIR method^9,40^. CMR image analysis was not performed for three participants due to technical issues with image acquisition.

### Catheter ablation

Catheter ablation procedures were performed following the institutional standard of care. Transfemoral and transseptal access was obtained, and left atrial pressures were recorded. A ten-electrode catheter (Inquiry, Abbott) was placed in the distal coronary sinus or great cardiac vein, and a second similar ten-electrode catheter was placed with its proximal electrodes at the high right atrium and its tip near the lateral cavotricuspid isthmus.

In patients who presented in sinus rhythm, a standardized AF induction protocol was performed using right atrial decremental burst pacing with eight-beat sequences, starting with a 300-ms pacing cycle length and decreasing to 200 ms (or the effective atrial refractory period) by steps of 10 ms.

A 3D electroanatomical map (CARTO mapping system, Biosense Webster) of the left atrium was obtained in AF using a 20-electrode circular mapping catheter (LASSO catheter, Biosense Webster). Although BV has been recognized as a surrogate for left atrial fibrosis independently of the underlying rhythm^41,42^, electrogram amplitudes depend markedly on many factors, such as physiological rate and wavefront dynamics, especially when obtained in sinus rhythm^43^. Mapping was obtained during AF as this practice exhibits the highest correlations between low voltage and left atrial fibrosis^44^. For eight patients, the map was obtained in sinus rhythm because sustained AF was not inducible or not induced. Complete left atrial anatomy (including the proximal pulmonary veins) was obtained by fast anatomical mapping (FAM, Biosense Webster), and a BV map was obtained by measuring the maximum bipolar peak-to-peak voltage amplitude during the annotation window.

In all participants, circumferential pulmonary vein isolation was performed (Thermocool SmartTouch SurroundFlow catheter, Biosense Webster) using a power range of 25–35 W and an application duration of 20–30 s titrated following standard practice. Pulmonary vein isolation (entrance block) was confirmed using the circular mapping catheter. Substrate modification of the extrapulmonary veins was performed at the operator’s discretion, targeting fractionated electrograms in the coronary sinus, left atrium and/or right atrium. Additionally, linear ablation lesions (for example, on the left atrial roof) were created at the operator’s discretion based on substrate distribution. Bipolar electrograms at baseline were filtered (band pass 30–500 Hz) and digitally recorded at a sampling frequency of 1,000 Hz (LabSystem Pro, Boston Scientific) along with surface electrocardiography.

### Computational fluid dynamics

The coordinates and measured variables of all data points recorded during the catheter ablation procedure were extracted and used to generate a patient-specific 3D model by using Poisson surface reconstruction (Fig. 1). Flow extensions were added on the pulmonary veins and the mitral valve with a length equal to ten times the equivalent diameter of each orifice to ensure that the flow is fully developed. The 3D model with the flow extensions was meshed with polyhedral elements and a five-element-wide prismatic boundary layer on all wall surfaces. The meshing parameters were kept constant for all cases, resulting in volumetric mesh sizes in the range of 0.9–1.7 million elements, depending on the size of the left atrium.

We performed a transient CFD simulation by running the model for six consecutive heart cycles to allow for the stabilization of the transient phenomena. The time step was fixed at 0.25 ms, which was sufficient for the solution to converge in all cases and time steps. The flow was assumed to be laminar, which was verified by checking the maximum Reynolds number. The blood was modeled as a Carreau–Yasuda fluid^45^. We imposed a patient-specific periodic velocity profile at the inlets, which was derived from the phase-contrast CMR image (Fig. 2a,b). If CMR imaging was performed during sinus rhythm, the profile was adapted by removing the mitral A-wave (second peak) to simulate AF, by ensuring that the first derivative remains continuous (Fig. 2a)^37^. This constitutes a good assumption when individual data for each pulmonary vein are not available, as was demonstrated by a similar computational analysis^31^. The measured profile also controlled the period of the simulation. At the outlet, we imposed the mean atrial pressure, as measured during the ablation procedure. The walls were assumed rigid due to the limited wall motion that occurs during AF^45^. A diffusion equation was added to the flow equations and a source term was added throughout the atrium to calculate the distribution of the BA throughout the atrial domain, as described previously^46^. The distribution of BA was measured at a distance of 1 mm from the atrial wall to avoid wall boundary effects. Second-order discretization schemes were selected for all the model variables. The models were solved in EPFL’s high-performance computing clusters (SCITAS), whereas the average computational time for each case was 30 h. The meshing and simulation steps were performed in Fluent (Ansys).

### Data postprocessing

We exported the flow variables of the last heart cycle of each CFD simulation. We used the exported time-dependent distribution of WSS on the atrial wall surface to calculate common indices of shear stress following the standard methodology^47,48^. Specifically, we calculated the TAWSS; the oscillatory shear index (OSI); the endothelial cell activation potential (ECAP); the relative residence time (RRT); the highly oscillatory, low-magnitude shear (HOLMES) index; and the time-averaged WSSG on the flow direction, as follows:

1 $${\mathrm{TAWSS}}=\frac{1}{T}{\int }_{t-T}^{T}\Vert {\mathbf{\uptau}}\Vert{\mathrm{d}}t$$

2 $${\mathrm{OSI}}=\frac{1}{2}\left(1-\frac{\Vert {\int }_{t-T}^{T}{\mathbf{\uptau}}{\mathrm{d}t}\Vert }{\mathrm{TAWSS}}\right)$$

3 $${\mathrm{ECAP}}=\frac{\mathrm{OSI}}{\mathrm{TAWSS}}$$

4 $${\mathrm{RRT}}=\frac{1}{\Vert \frac{1}{T}{\int }_{t-T}^{T}{\mathbf{\uptau}}{\mathrm{d}}t\Vert }$$

5 $${\mathrm{HOLMES}}={\mathrm{TAWSS}}(0.5-{\mathrm{OSI}})$$

6 $${\mathrm{WSSG}}=\frac{1}{T}{\int }_{t-T}^{T}\frac{{\mathbf{\uptau}}\times {\mathbf{g}}}{\Vert {\mathbf{\uptau}}\Vert }{\mathrm{d}}t$$

where *T* is the heart period, *t* is the time, **τ** is the vector of the WSS and **g** is the vector of the projection of the WSSG of the flow direction^49^. These wall shear indices have been developed initially for computational studies on atherosclerosis and aneurysms, but they have shown great precision in localizing regions prone to structural and functional changes. The combination of high WSS and positive WSSG has been shown to induce internal elastic lamina damage, aneurysm formation and mural cell-mediated remodeling, whereas low WSS combined with high OSI can cause an inflammatory response on the aneurysmal wall^6,49,50^. The HOLMES index was proposed to quantify in a single index the combined effect of low but highly oscillatory wall shear^51^. Similarly, ECAP was suggested as an alternative index to localize regions with high OSI and low WSS by quantifying the susceptibility to thrombus formation^52^. The RRT is strongly correlated with OSI and serves as an index of near-wall blood stagnation^53^; however, despite its name, it remains a wall shear index that differs from BA, which is directly measured using the previously described methodology.

Consequently, we transferred the measured values of BV and IIR on the atrial wall surface used for the CFD simulation to allow for a quantitative comparison among the measured and calculated variables (Fig. 1). This was achieved by first aligning the CMR segmentation with the CFD surface mesh, using an iterative closest point algorithm, and then by interpolating the IIR and BV values on the CFD mesh using 3D radial basis function interpolation. The CFD mesh was chosen as the basis of this transform because most variables had already been calculated on it. This procedure resulted in a set of points with their corresponding BV, IIR and wall shear indices for each participant, and these values were used for the subsequent statistical analysis.

To create the 2D maps, we standardized the CFD mesh by clipping the pulmonary veins to a fixed length of 10 mm; then, it was registered to an anatomical atlas of the left atrium (Fig. 2c). The registered mesh was unfolded by constraining the mitral valve to the perimeter of a predefined 2D pattern to create the 2D maps of every variable and automatically divide the atrial wall into 24 regions (Fig. 2d). The unfolding process has been previously described in detail^54^, whereas the 2D maps serve as an equivalent of the standardized bull’s-eye diagrams that are used for the left ventricle. We calculated the mean value of each variable per region of the 2D atrial map, $\bar{{x}_{i}}$, as the arithmetic mean and the normalized mean value per region as ${{\bar{x}}_{{\textrm{n}},i}}={{\bar{x}}_{i}}/\max ({{\bar{x}}_{i}})$, where *x* is the variable, *x*_n_ the normalized variable and *i* the region number. The postprocessing pipeline was automated by requiring minimal user input and was performed using in-house algorithms programmed in Python (Python Software Foundation) and MATLAB (MathWorks). These algorithms are available on GitHub (https://github.com/g-rov/lausm).

### Statistical analysis

Categorical variables are expressed as percentages. Continuous variables are expressed as means ± s.d. unless differently mentioned. Correlations between shear stress indices, BV and IIR were assessed individually for each case using the Pearson correlation coefficient. TAWSS values for each case were further grouped into quartiles (Q1–Q4). For categorical variables, comparisons among groups were performed using the chi-square test. For continuous variables, comparisons were performed with analysis of variance (ANOVA) if the data were normally distributed; otherwise, the Kruskal–Wallis test was used. Normality was assessed with the Shapiro–Wilk test. Levene’s test was used to assess the homogeneity of variance among the compared groups, and in case of violation, Welch’s ANOVA test was used. The Fisher *z*-transformation was applied to the correlation coefficients to allow statistical comparisons. Statistical significance was assumed at a two-sided *P* value of 0.05. Statistical analysis was performed in IBM SPSS Statistics (released 2020, IBM SPSS Statistics for Windows, version 27.0, IBM).

### Reporting summary

Further information on research design is available in the Nature Portfolio Reporting Summary linked to this article.

## Supplementary information

Reporting Summary

## Source data

Source Data Fig. 3 Statistical source data.

Source Data Fig. 7 Statistical source data.

Source Data Extended Data Fig. 1 Statistical source data.

Source Data Extended Data Fig. 2 Statistical source data.

## Data Availability

The data supporting the findings of this study are available via figshare at 10.6084/m9.figshare.26068273 (ref. ^55^). Source data are provided with this paper.

## Code availability

The code used in this article is available via GitHub at https://github.com/g-rov/lausm and a snapshot of the code is available via figshare at 10.6084/m9.figshare.26068273 (ref. ^55^).
