# Supplementary material for: Left atrial wall shear stress correlates with fibrosis in patients with atrial fibrillation
Source: Nat Cardiovasc Res. 2025 May 13;4(6):677–88. doi: 10.1038/s44161-025-00651-z (PMC12170334; doi:10.1038/s44161-025-00651-z)
Supplement: Supplementary file 1 — Reporting Summary [file 44161_2025_651_MOESM1_ESM.pdf]

nature portfolio

Corresponding author(s): Dionysios Adamopoulos

Last updated by author(s): Mar 30, 2025

Reporting Summary

Nature Portfolio wishes to improve the reproducibility of the work that we publish. This form provides structure for consistency and transparency in reporting. For further information on Nature Portfolio policies, see our Editorial Policies and the Editorial Policy Checklist.

Statistics

For all statistical analyses, confirm that the following items are present in the figure legend, table legend, main text, or Methods section.

n/a	Confirmed	
☐	☒	The exact sample size (*n*) for each experimental group/condition, given as a discrete number and unit of measurement
☐	☒	A statement on whether measurements were taken from distinct samples or whether the same sample was measured repeatedly
☐	☒	The statistical test(s) used AND whether they are one- or two-sided<br>*Only common tests should be described solely by name; describe more complex techniques in the Methods section.*
☒	☐	A description of all covariates tested
☐	☒	A description of any assumptions or corrections, such as tests of normality and adjustment for multiple comparisons
☐	☒	A full description of the statistical parameters including central tendency (e.g. means) or other basic estimates (e.g. regression coefficient) AND variation (e.g. standard deviation) or associated estimates of uncertainty (e.g. confidence intervals)
☐	☒	For null hypothesis testing, the test statistic (e.g. *F*, *t*, *r*) with confidence intervals, effect sizes, degrees of freedom and *P* value noted<br>*Give P values as exact values whenever suitable.*
☒	☐	For Bayesian analysis, information on the choice of priors and Markov chain Monte Carlo settings
☒	☐	For hierarchical and complex designs, identification of the appropriate level for tests and full reporting of outcomes
☐	☒	Estimates of effect sizes (e.g. Cohen's *d*, Pearson's *r*), indicating how they were calculated

Our web collection on statistics for biologists contains articles on many of the points above.

Software and code

Policy information about availability of computer code

Data collection	- IMRIS Invision, version 11E, commercial, Siemens Erlangen, DE, used for the CMR acquisition with a 3-T MRI scanner (MAGNETOM Skyra, Siemens Erlangen,DE) in a multiroom Invision Surgical Theater.<br>- CARTO mapping system, version 7.1.80.33, commercial, Biosense Webster, Irvine, California, USA, used for 3D electro-anatomical mapping<br>- 3D Slicer, version 4.11, open-source, used for CMR reconstruction and segmentation.
Data analysis	- Ansys Suite, version 2021 R2, commercial, used for meshing and CFD.<br>- CloudCompare, version 2.13.2, open source, used for Iterative Closest Point Algorithm.<br>- SciPy, version 1.15.2, open source, used for the 3D Radial Basis Function interpolation.<br>- IBM SPSS statistics, Version 27.0, commercial, IBM Corp. ,Armonk, NY, used for statistical analyses.<br>- Custom, open-source, post-processing code, programmed in Python (v. 3.6 & 3.12) and Matlab (v. 2024a). These algorithms are available on github.com/g-rov/lausm.

For manuscripts utilizing custom algorithms or software that are central to the research but not yet described in published literature, software must be made available to editors and reviewers. We strongly encourage code deposition in a community repository (e.g. GitHub). See the Nature Portfolio guidelines for submitting code & software for further information.

Data

Policy information about availability of data

All manuscripts must include a data availability statement. This statement should provide the following information, where applicable:

- Accession codes, unique identifiers, or web links for publicly available datasets
- A description of any restrictions on data availability
- For clinical datasets or third party data, please ensure that the statement adheres to our policy

The data supporting the findings of this study are available doi.org/10.6084/m9.figshare.26068273.

Research involving human participants, their data, or biological material

Policy information about studies with human participants or human data. See also policy information about sex, gender (identity/presentation), and sexual orientation and race, ethnicity and racism.

Reporting on sex and gender	Only sex was collected and reported in this study. No sex/gender analyses are applicable to our analyses.
Reporting on race, ethnicity, or other socially relevant groupings	No socially relevant groupings are applicable.
Population characteristics	15 patients (13 males, mean age 61±11 years)<br>All presented symptomatic episodes of AF with a clinical indication for catheter ablation. 10 subjects presented with paroxysmal and 5 with persistent AF, while 3 presented additionally atrial flutter.
Recruitment	All patients underwent a baseline echocardiography and only patients without hemodynamically significant (more than moderate) mitral regurgitation were eligible for the study. Furthermore, a condition for participation was the absence of contraindications to the CMR exam (e.g., metallic implants) and the participants' overall health, which had to allow them to undergo an extended CMR exam. Globally, no self-selection or other biases were identified in the recruitment process that could impact the results.
Ethics oversight	An informed written consent was obtained by each participant for use, analysis and publication of data in anomymized format. The Commission Cantonale d'éthique de la Recherche sur l'être humain (CCER) of the Canton of Geneva gave ethical approval for this work, approval number: 2023-02314. The participants did not receive any compensation for participating in the study.

Note that full information on the approval of the study protocol must also be provided in the manuscript.

Field-specific reporting

Please select the one below that is the best fit for your research. If you are not sure, read the appropriate sections before making your selection.

☒ Life sciences ☐ Behavioural & social sciences ☐ Ecological, evolutionary & environmental sciences

For a reference copy of the document with all sections, see nature.com/documents/nr-reporting-summary-flat.pdf

Life sciences study design

All studies must disclose on these points even when the disclosure is negative.

Sample size	n=15. Prospective recruitment of all patients that meet the inclusion/exclusion criteria. No sample size calculations were performed, because most analyses are within-subject. Additionally, due to the method followed, from each subject approximately 25,000 points are generated, creating a sufficient sample size for even small differences. For the comparison between AF types, we did not expect to find any difference, given the large interpersonal variability. For the comparison among regions, we expected the blood age to be multiple times higher in certain regions (e.g. appendage) compared to the pulmonary veins, hence a small sample size would suffice.<br>Finally, the nature of the present study was exploratory, with the primary aim of identifying patterns or correlations rather than generalizing the findings to a larger population. The consistency of the results though across our small group of patients enhances the reliability of the observed associations and lays the groundwork for future research.
Data exclusions	No data were excluded from this study.<br>Patients were excluded if they had hemodynamically significant (more than moderate) mitral regurgitation. This was necessary because additional data which were not collected would be required for the computational model (CFD) and the CFD model itself would require adaptation.
Replication	The electro-anatomical mapping (catheterization) is operator-dependent but reproducibility cannot be assessed due to ethical concerns.<br>The MRI segmentation was performed using standard methodology with a clearly defined and reproducible strategy.<br>The CFD pipeline is deterministic and reproducible as its parameters were clearly defined.

	The post-processing pipeline is fully automated and deterministic, hence fully reproducible.<br>For the MRI, CFD and post-processing pipeline replicates were not performed as they were considered unnecessary.
Randomization	This is not relevant to our study, as there is only one group, with all its members receiving the same treatment.
Blinding	The operators that performed the measurements (catheterization, CMR) and segmentation were blinded to the results of the CFD and following analyses.<br>Blinding in terms of group allocation is not applicable, as there was only one study group.

Reporting for specific materials, systems and methods

We require information from authors about some types of materials, experimental systems and methods used in many studies. Here, indicate whether each material, system or method listed is relevant to your study. If you are not sure if a list item applies to your research, read the appropriate section before selecting a response.

Materials & experimental systems

n/a	Involved in the study
☒	☐ Antibodies
☒	☐ Eukaryotic cell lines
☒	☐ Palaeontology and archaeology
☒	☐ Animals and other organisms
☒	☐ Clinical data
☒	☐ Dual use research of concern
☒	☐ Plants

Methods

n/a	Involved in the study
☒	☐ ChIP-seq
☒	☐ Flow cytometry
☒	☐ MRI-based neuroimaging

Plants

Seed stocks	N/A
Novel plant genotypes	N/A
Authentication	N/A
